# Solving the diagnostic dilemma in bone infections: metagenomic next generation sequencing enhances pathogen identification accuracy

**DOI:** 10.3389/fmed.2025.1699607

**Published:** 2026-01-07

**Authors:** Jiheng Xiao, Congli Pu, Xianglong Zhou, Xiaokang Zhang, Shanshan Zhang, Puxin Yang, Yingze Zhang, Liming Xiong

**Affiliations:** 1 Department of Orthopaedics, Union Hospital, Tongji Medical College, Huazhong University of Science and Technology, Wuhan, Hubei, China; 2 Department of Orthopaedic Surgery, Third Hospital of Hebei Medical University, Shijiazhuang, Hebei, China; 3 NHC Key Laboratory of Intelligent Orthopaedic Equipment, Third Hospital of Hebei Medical University, Shijiazhuang, Hebei, China; 4 Department of Gynecological Oncology, Fudan University Shanghai Cancer Center, Fudan University, Shanghai, China; 5 School of Medicine, Nankai University, Tianjin, China

**Keywords:** mNGS, bone infection, diagnostic sensitivity, pathogen detection, prognosis

## Abstract

**Objective:**

Metagenomic Next Generation Sequencing (mNGS) offers a rapid, unbiased, and culture-independent approach to pathogen identification by analyzing all nucleic acids present in clinical samples. Despite its growing use, the diagnostic utility of mNGS in bone infections remains inadequately characterized. This study aimed to assess the diagnostic accuracy of mNGS compared to conventional microbial cultures and to explore its associations with clinical severity and patient outcomes.

**Methods:**

We retrospectively enrolled 135 adult patients treated for suspected bone infections between October 2023 to January 2025 at Union Hospital, Tongji Medical College. Among these, 101 patients were classified as the infection group (IG) based on clinical and laboratory criteria, encompassing osteomyelitis, post-traumatic limb infections, and diabetic foot infections. mNGS results were compared to traditional cultures in terms of sensitivity, specificity, predictive values, and discordant cases. The IG was further stratified into mNGS-positive (*n* = 95) and mNGS-negative (*n* = 6) subgroups. Clinical parameters—including leukocyte differentials, C-reactive protein (CRP), procalcitonin (PCT), albumin, length of hospital stay, and mortality—were analyzed in relation to mNGS findings.

**Results:**

Among all patients, 74.81% were confirmed to have infections. mNGS demonstrated a markedly higher sensitivity than culture (94.06% vs. 47.52%, *p* = 0.000) while maintaining comparable specificity (85.29% vs. 76.47%, *p* = 0.549). Age showed a potential trend in influencing mNGS positivity (*p* = 0.092). Although not statistically significant, mNGS-positive patients tended to have longer hospitalizations (*p* = 0.098), suggesting possible associations with infection complexity or pathogen load.

**Conclusion:**

mNGS substantially enhances the diagnostic yield for bone infections, particularly in polymicrobial, low-abundance, or culture-negative scenarios. mNGS-negative patients had significantly shorter hospital stays and a lower rehospitalization rate. Its rapid and comprehensive pathogen detection may enable more timely and targeted antimicrobial therapy, potentially improving patient outcomes and reducing healthcare burden. These findings support the integration of mNGS as a valuable adjunct to conventional diagnostic workflows in orthopedic infectious diseases.

## Introduction

Infections remain a prominent contributor to morbidity and mortality worldwide (1, 2). In recent years, the misuse of antimicrobial drugs has resulted in significant alterations in pathogenic microbial species and their resistance, profoundly impacting patient prognosis in clinical settings (3). Accurately identifying pathogenic microorganisms poses a crucial challenge for clinicians. Orthopedic infections are common complications in orthopedic practice, primarily characterized by postoperative wound infections and mixed infections in open injuries, often exhibiting high levels of drug resistance (4–6). These infections not only disrupt local tissue blood flow and healing but also impede the recovery of bone, joint, and muscle function, thereby increasing the physical, mental, and economic burden on patients (7). While the traditional microbiological culture method serves as the “gold standard” for diagnosing bone infections due to its large sample size and affordability (8), its sensitivity and diagnostic speed are compromised by factors such as bacterial biotypes (9). Even when bacterial cultures yield positive results, the prolonged incubation period may result in patients missing the optimal window for treatment, thereby negatively impacting therapeutic outcomes. Specific culture techniques are necessary for certain bacterial infections, particularly those with low virulence or prior antibiotic exposure, where false-negative results may occur (10), thereby complicating accurate diagnosis. Culture-independent methods, such as serological tests and nucleic acid amplification assays (11), hold promise for expanding pathogen detection. Consequently, there is an urgent need to develop novel diagnostic approaches that enable the rapid and precise identification of the microbial pathogens responsible for bone infections.

Metagenomic next-generation sequencing (mNGS) is an advanced diagnostic tool that enables direct sequencing of clinical samples to identify a broad range of pathogens, including bacteria, fungi, viruses, and parasites (12, 13). With ongoing advancements in mNGS technology and an increasing number of clinical studies, its application in diagnosing infectious diseases has expanded (14). The first reported case of a central nervous system infection diagnosed using mNGS occurred at the University of California in 2014 (15), marking a pivotal milestone in its clinical adoption. One of the primary advantages of mNGS is its ability to perform high-throughput sequencing of nucleic acids from all microorganisms present in clinical samples, allowing for unbiased detection and comparison of microbial species and sequences (16). Unlike traditional culture methods, mNGS does not rely on microbial cultivation, enabling more accurate identification and typing of pathogens through the direct extraction of nucleic acids (17–19). While mNGS has demonstrated significant promise in detecting pathogens in various clinical specimens, such as bronchoalveolar lavage fluid, blood, and cerebrospinal fluid, its application is not without limitations, including the potential for false positives and negatives, which necessitate careful interpretation and result confirmation (20–22). However, research on the application of mNGS specifically to clinical tissue samples from bone infections remains relatively limited, particularly regarding the correlation between its findings and patient outcomes.

The primary objective of this study was to evaluate the diagnostic performance of mNGS against conventional culture in a cohort of patients with suspected bone infections. We aimed not only to compare their sensitivity and specificity but also to investigate the potential of mNGS results to reflect clinical severity and influence patient prognosis (23).

## Materials and methods

### Study design and patient selection

**Figure 1:**
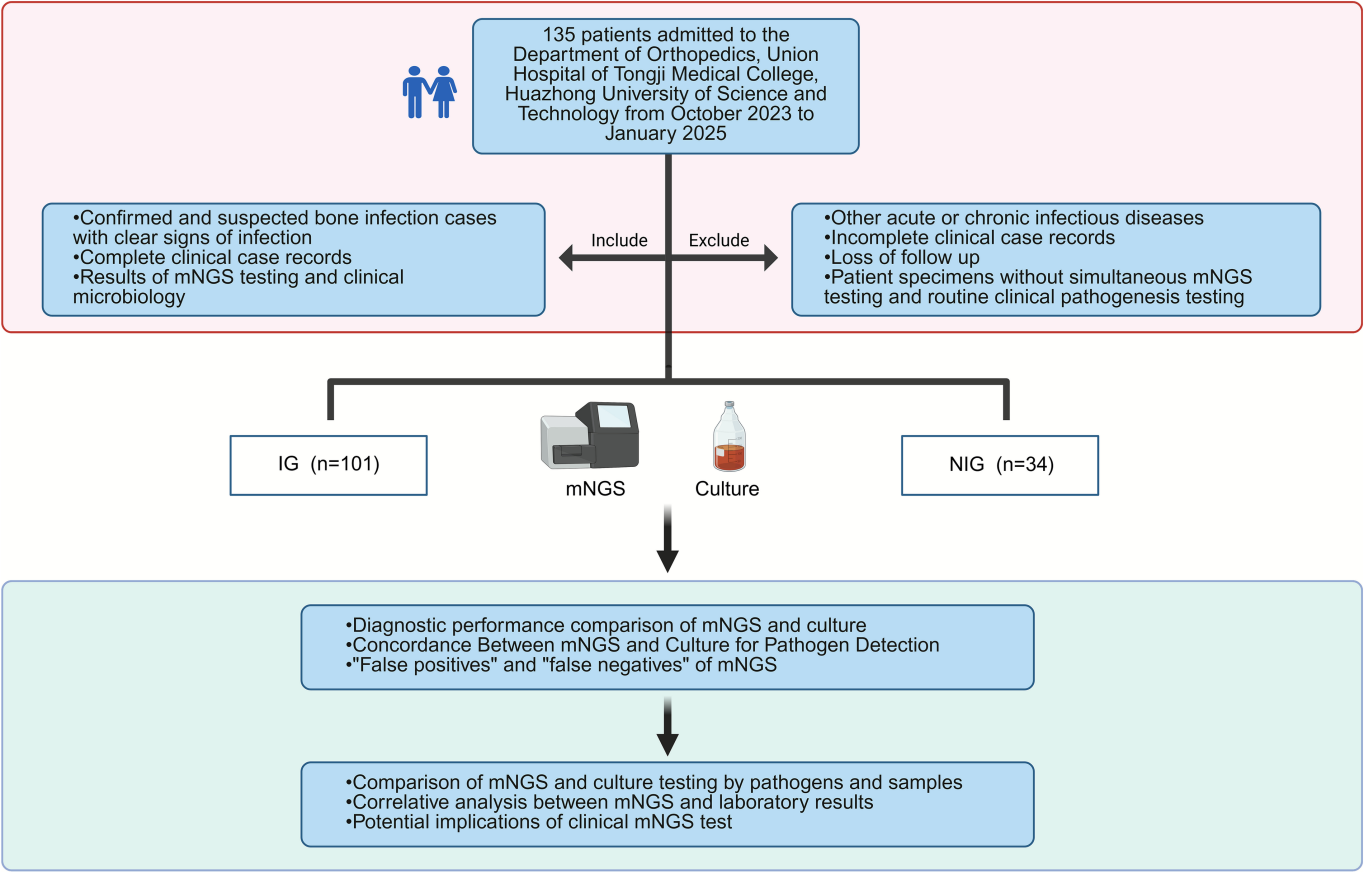
Flowchart of sample selection, classification, and comparison analysis. Orthopedic disease-related cases were categorized into the infectious group (IG) and non-infectious group (NIG).

The clinical study retrospectively analyzed the clinical information of 135 patients admitted to the Department of Orthopedics, Union Hospital of Tongji Medical College, Huazhong University of Science and Technology from October 2023 to January 2025, including 101 infected patients who underwent mNGS testing. The final diagnosis for group assignment was established by a panel of senior orthopedists and infectious disease specialists, based on a comprehensive review of all available clinical, laboratory, imaging, and pathological data, and discharge diagnosis. According to the final clinical diagnosis, 101 cases were divided into the infectious disease group (IG) and 34 cases in the non-infectious disease group (NIG). The specimens were tested by mNGS (BGI, China) and clinical microbiology, and the final diagnosis was made by the patients’ relevant test reports and clinical presentation. Meanwhile, biochemical indexes, including total albumin, C-reactive protein (CRP), procalcitonin (PCT), fasting glucose, and triglycerides, were collected. The flow diagram of case inclusion and exclusion was shown in Figure 1. The following inclusion criteria were used: (1) Confirmed and suspected bone infection cases with clear signs of infection; (2) Complete clinical case records; (3) Results of mNGS testing and clinical microbiology. The following are exclusion criteria: (1) Other acute or chronic infectious diseases; (2) Incomplete clinical case records; (3) Loss of follow-up; (4) Patient specimens without simultaneous mNGS testing and routine clinical pathogenesis testing. The Ethics Committee of the Union Hospital of Tongji Medical College of Huazhong University of Science and Technology approved the study ([2024] Ethics No. (0904), MR-42-24-047721).

### Data collection and the declaration of Helsinki statement

Clinical data were collected independently by two experienced attending physicians, including demographics, clinical manifestations, clinical sample types, laboratory examination, treatment methods, outcomes, and prognosis. According to the final diagnosis and outcome, the patients were divided into the infectious disease group and the non-infectious disease group. All human specimens and data included in this study comply with the Declaration of Helsinki and adhere to the ethical guidelines for medical research involving human subjects.

### mNGS methodology and analysis

#### Sample collection and pretreatment

**Figure 2:**
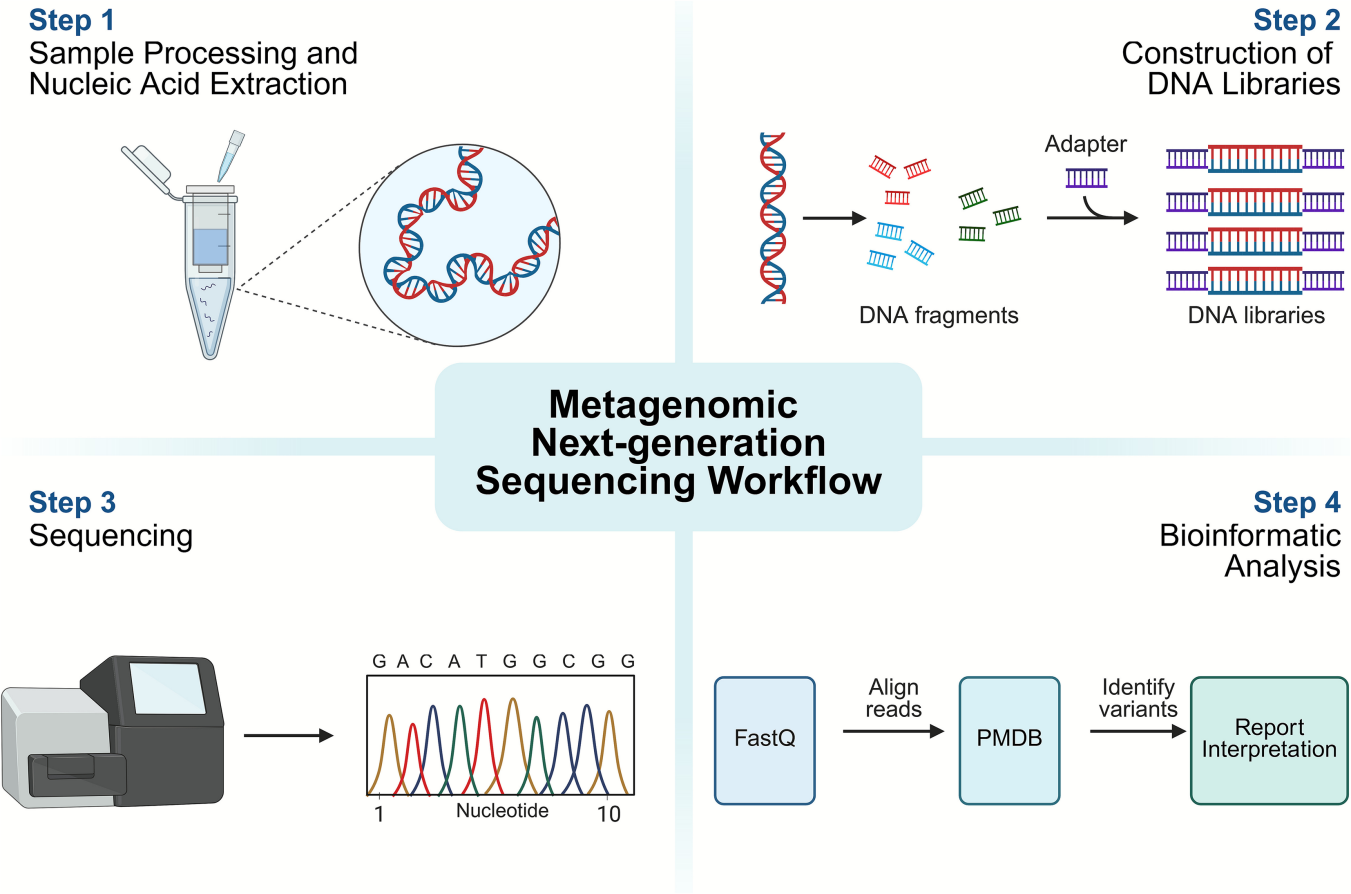
Workflow of metagenomic next-generation sequencing and analysis.

All specimens were collected from the clinically identified site of suspected bone infection prior to antimicrobial administration to avoid false-negative results. Sampling was performed by experienced orthopedic surgeons to ensure consistency and target the actual infectious focus. Figure 2 illustrates in detail that all samples underwent meticulous processing through a four-step method. The specific procedures for each sample type were as follows:

Fresh tissue samples (*n* = 22): Soybean-sized tissue blocks were surgically excised from the core infectious lesion, identified by the presence of inflammation, necrotic bone, or purulent material. These blocks were rinsed twice with sterile normal saline to remove surface contaminants. Samples were then cut into 1–2 mm^3^ fragments using sterile scissors and homogenized in a tissue grinder at 60 Hz for 2 min to disrupt the extracellular matrix. The homogenate was transferred to a 1.5 mL microcentrifuge tube, mixed with 600 μL lysis buffer and 250 μL glass beads (0.1 mm diameter), and vortexed at 3,000 rpm for 30 min. Subsequently, 7.2 μL lysozyme (20 mg/mL, Sigma-Aldrich) was added, and the mixture was incubated at 37 °C for 45 min to enhance lysis of Gram-positive bacteria and fungi with thick cell walls.

Intraoperative pus samples (*n* = 56): Pus swabs were collected directly from abscess cavities or deep wound pockets during surgical debridement. Pus swabs were eluted in 1 mL sterile PBS by vortexing at 2,500 rpm for 10 min. The eluate was centrifuged at 8,000 g for 5 min to pellet pathogens and remove supernatant containing necrotic debris and mucin. The pellet was resuspended in 600 μL lysis buffer, mixed with 250 μL glass beads, and vortexed at 2,800 rpm for 25 min. Lysozyme (7.2 μL) was added, followed by incubation at 37 °C for 30 min. This centrifugation step reduces interference from non-microbial nucleic acids, which is critical for detecting polymicrobial infections.

Wound secretion samples (*n* = 23): Secretions (≥500 μL) were aseptically aspirated using sterile syringes from the base of deep wounds or sinuses after superficial cleaning with saline to minimize contamination from skin colonizers. For viscous secretions, 100 μL DNase-free RNase (1 mg/mL, Thermo Fisher) was added to degrade extracellular nucleic acids from dead microbes. The concentrated sample was mixed with 600 μL lysis buffer and 250 μL glass beads, vortexed at 3,200 rpm for 30 min, and incubated with 7.2 μL lysozyme at 37 °C for 35 min. Viscous secretions are prone to false negatives due to low pathogen load, so vigorous vortexing was used to improve nucleic acid release.

#### DNA extraction and quality control

After pretreatment, 0.3 mL of each sample was transferred to a new tube, and total DNA was extracted using the TIANamp Micro DNA Kit (DP316, TIANGEN BIOTECH) following the manufacturer’s instructions. DNA concentration and purity were quantified using a NanoDrop 2000 (Thermo Scientific). Only samples with DNA concentration ≥10 ng/μL and A260/A280 ratio 1.8–2.0 were used for library construction, as low-quality DNA significantly reduces mNGS sensitivity.

#### Construction of DNA libraries and sequencing

DNA libraries were constructed through DNA fragmentation, end-repair, adapter ligation, and PCR amplification. Agilent 2100 was used for quality control of the DNA libraries. Quality-qualified libraries were pooled, a DNA Nanoball (DNB) was made, and sequenced by the BGISEQ-50/MGISEQ-2000 platform (24).

#### Bioinformatic analysis

High-quality sequencing data were generated by removing low-quality reads, followed by computational subtraction of human host sequences mapped to the human reference genome (hg19) using Burrows-Wheeler Alignment (25). The remaining data, after removal of low-complexity reads, were classified by simultaneously aligning to the Pathogens metagenomics Database (PMDB), consisting of bacteria, fungi, viruses, and parasites. PMseq high-throughput genetic test achieves comprehensive coverage of 17,500 human-associated pathogenic microorganisms. The classification reference databases were downloaded from NCBI (ftp://ftp.ncbi.nlm.nih.gov/genomes/) (26).

#### Criteria for a positive mNGS results

Types of pathogenic microorganisms include bacteria, fungi, viruses, parasites, *mycobacterium tuberculosis*, mycoplasma, chlamydia, and rickettsia. Multiple parameters were obtained from the sequencing platform, including the number of specifically mapped reads, sequence counts, and abundance at the level of all microbial species and genera, and to detect and match background microorganisms, better excluding human colonizing microbial confounders. Considering confounding factors such as nucleic acid contamination, total number of sequencing reads, and pathogen genome size, coverage was used as a measurement parameter in this study (27, 28).

(1) Top 10 pathogens by sequencing coverage: referring to identifying the ten pathogens with the highest sequencing coverage rate among all the detected pathogens. Coverage rate is a measure of how well the DNA of each pathogen is represented in the sequencing data, reflecting the proportion of reads mapped to each pathogen’s genome.
(2) Strictly mapped reads greater than three for Top 10 Pathogens: for each of the top 10 pathogens, this criterion states that the number of strictly mapped reads (reads that align with high specificity to the pathogen’s genome) must be greater than three, ensuring that there is sufficient evidence to confidently identify the presence of the pathogen and reduces the risk of false positives caused by low-level contamination or non-specific alignment.
(3) Furthermore, the determination of a clinically relevant pathogen from the list of microorganisms identified by mNGS involved a multifaceted interpretation process, rather than reliance on a single metric. The process must be considered: The inherent pathogenicity of the microorganism. Well-known pathogens were weighted more heavily than common commensals or environmental organisms. The abundance of microbial sequences, with higher coverage and read counts, increases the confidence in a true positive result. Most critically, the clinical context. The mNGS findings were evaluated against the patient’s presentation, radiological imaging, and standard inflammatory markers. A microorganism was considered causative only if its presence was consistent with the clinical picture of an active infection, and no other more plausible non-infectious diagnosis could explain the presentation.

#### Adjudication criteria for discordant mNGS results

To ensure an objective and clinically contextualized interpretation of mNGS findings, particularly for positive results in the Non-Infection Group (NIG), a set of predefined adjudication criteria was established and applied by an independent panel of two senior orthopedists and one clinical microbiologist. The classification of a positive mNGS result as a potential false-positive was based on the fulfillment of at least one of the following objective criteria:

Clinical and radiological evidence: The patient lacked conclusive clinical signs and symptoms or radiological evidence suggestive of an active infection attributable to the pathogen identified by mNGS. Furthermore, a definitive alternative, non-infectious diagnosis, such as aseptic inflammatory reaction, pathologic fracture, or implant failure, was established that fully explained the patient’s presentation.

Microbiological corroboration: The mNGS-identified pathogen was not confirmed by any other microbiological method, such as culture or serology, performed on the same or contemporaneously collected samples, and there was no prior clinical documentation of an active infection with that pathogen.

Treatment response evidence: In cases where empiric or targeted antimicrobial therapy was administered against the mNGS-identified pathogen, no subsequent clinical improvement was observed, as assessed by persistent symptoms and unchanged inflammatory markers (CRP, PCT).

This adjudication process aligns with the recommended framework for interpreting mNGS results in complex clinical scenarios, emphasizing that the clinical significance of a detected microorganism is ultimately determined by its consistency with all available patient data rather than the detection alone.

#### Adjudication of mNGS results and therapeutic adjustments

For each patient, the mNGS report was reviewed by a multidisciplinary team including orthopedists, infectious disease specialists, and clinical microbiologists. The clinical relevance of each detected microbe was assessed based on the following: (1) the number of strictly mapped reads and coverage relative to the background; (2) the patient’s clinical presentation and immune status; (3) supporting laboratory and imaging findings; (4) whether the microbe was recognized as a common pathogen in bone infections. Therapeutic adjustments were made if the detected pathogens were deemed clinically significant and not adequately covered by the current regimen. For bacteria and fungi, antimicrobial selection was based on known local susceptibility patterns or, when available, subsequent culture and susceptibility testing. For viruses, specific antiviral therapy was initiated. The dosing and duration of therapy were determined by the type of infection, the identified pathogens, and the patient’s clinical response, following established guidelines.

#### Statistical analysis

Data were analyzed using SPSS 22.0 software. Categorical variables, such as rehospitalization rate, were presented as numbers and percentages (n, %). Comparisons between groups for these variables were performed using the Pearson χ^2^ test or Fisher’s exact test, as appropriate. For continuous variables that were not normally distributed, such as hospital days and operation time, data were presented as median with interquartile range (IQR), and comparisons between groups were conducted using the Mann–Whitney U test for two groups or the Kruskal-Wallis H test for multiple groups, as in the comparison of different bone infection diseases. *p* values < 0.05 were considered significant, and all tests were two-tailed.

## Results

### Sample and patient characteristics

**Figure 3:**
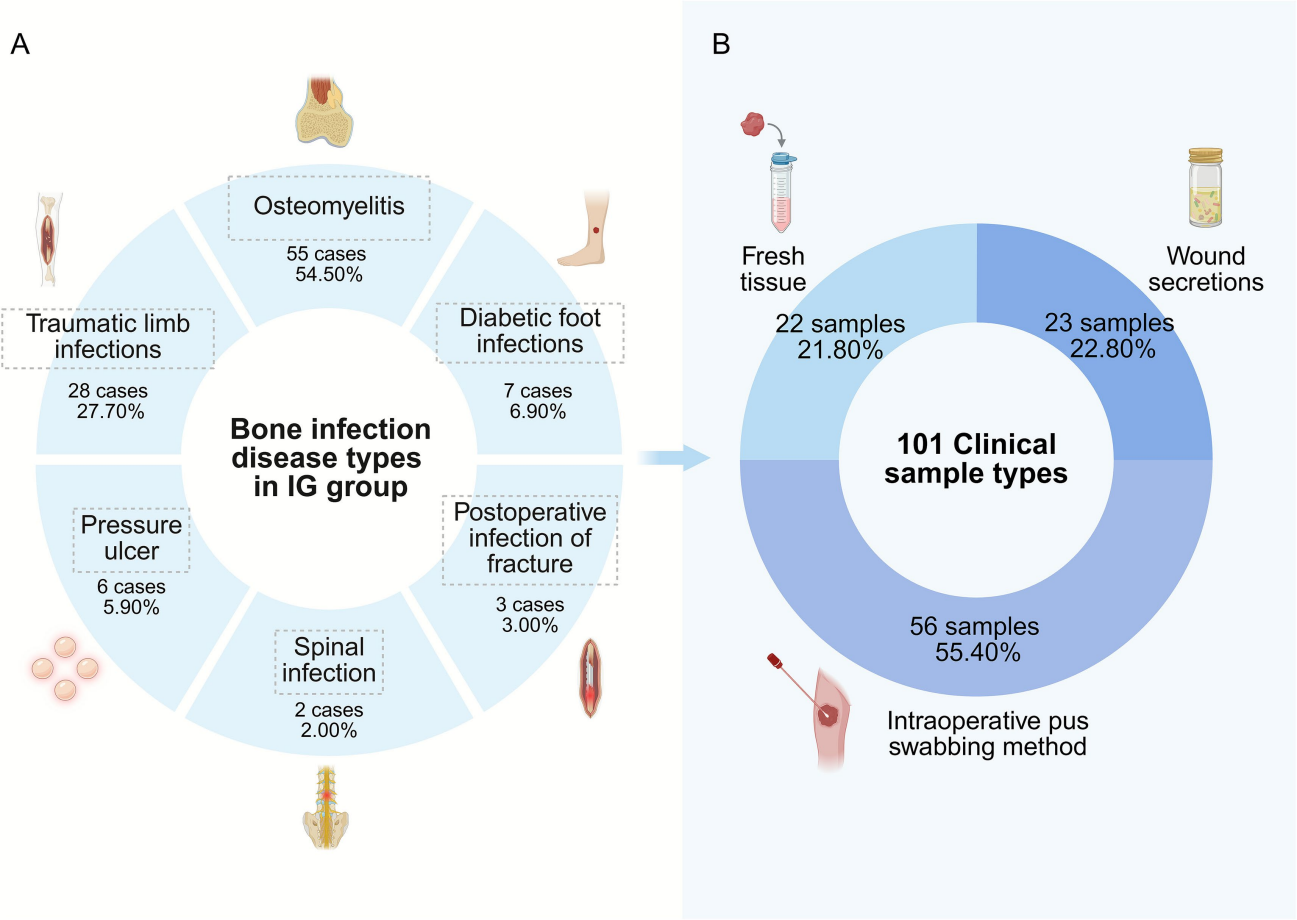
Types of bone infections in the 101 infected group of patients and the distribution of sampling types at infected tissue sites. **(A)** Distribution of bone infection disease types in the IG Group. The pie chart illustrates the proportion of different bone-related infection diagnoses among the 101 cases in the IG group, with osteomyelitis being the most prevalent (55 cases, 54.50%). **(B)** Distribution of clinical sample types collected. This chart shows the types of 101 clinical specimens obtained from IG group for analysis, with intraoperative pus swabs constituting the majority (56 samples, 55.40%).

The basic demographic characteristics of the patients are shown in Table 1. There were 107 men and 28 women, with a mean age of 56.53 years, and a mean hospital stay of 21.34 days. The 135 patients were divided into infected and non-infected groups, and there were no significant differences between the two groups in terms of the proportions of age, sex, and hospitalization days (*p* = 0.193, 0.960, and 0.051). All of them were orthopedically infected in this study, in the IG (101/135 [74.81%]), as shown in Figure 3A, the majority of patients were diagnosed with osteomyelitis (55/101 [54.50%]), followed by traumatic limb infections (28/101 [27.70%]) and diabetic foot infections (7/101 [6.90%]). The 101 clinical samples were divided into three types according to the sampling method, and the distribution of types is shown in Figure 3B, where wound secretions in 23 cases (22.80%), intraoperative pus swabbing method in 56 cases (55.40%), and fresh tissue in 22 cases (21.80%).

**Table 1:** Demographic characteristics of samples.

Characteristics	Total	IG	NIG	*P* value
Sample amount	135	101	34	–
Age (years)	56.53 (15–79)	60.62	55.15	0.193
Sex, *n* (%)				
Male	107 (79.25)	80 (79.20)	27 (79.41)	0.960
Female	28 (20.74)	21 (20.8)	7 (20.58)	
Hospital day, median (IQR)	21.34 (15–29)	23.64 (21–26)	20.27 (20–24)	0.051

### Diagnostic performance comparison of mNGS and culture

#### Comparison of diagnostic performance for differentiating IG from NIG

**Figure 4:**
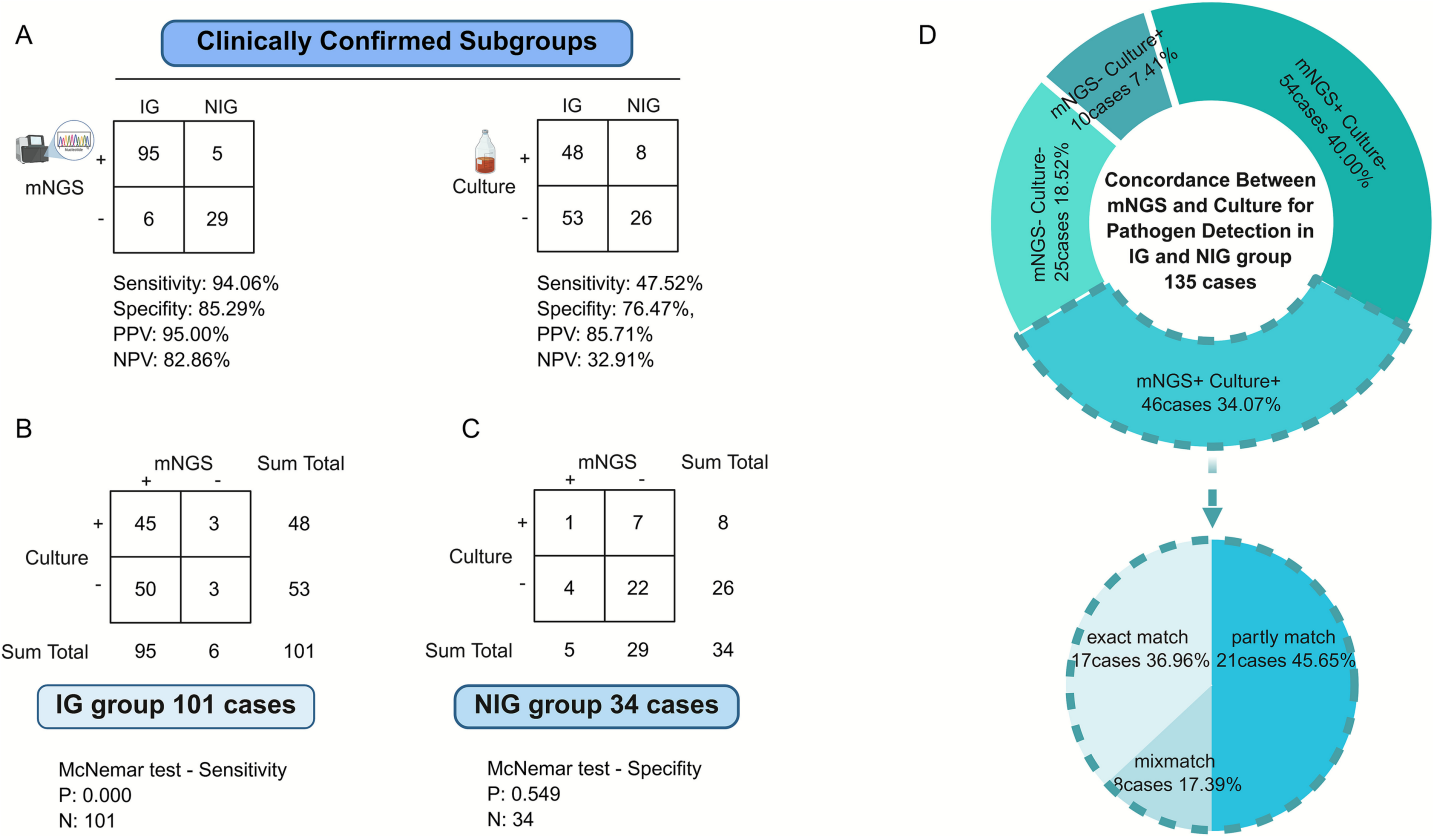
Comparison of diagnostic efficacy and concordance between mNGS and conventional culture. **(A)** Diagnostic performance comparison against clinical confirmation. Contingency tables and diagnostic metrics (Sensitivity, Specificity, PPV, NPV) for mNGS and culture methods compared to the clinically confirmed subgroups (Infection Group, IG; Non-Infection Group, NIG). mNGS demonstrated superior sensitivity (94.06% vs. 47.52%) and NPV (82.86% vs. 32.91%) compared to culture. **(B)** Consistency between mNGS and culture in the Infection Group (IG). Contingency table comparing detection results of mNGS and culture methods within the 101 IG cases. McNemar’s test indicates a statistically significant difference in sensitivity between the two methods (*p* < 0.001). **(C)** Consistency between mNGS and culture in the Non-Infection Group (NIG). Contingency table comparing detection results of mNGS and culture methods within the 34 NIG cases. McNemar’s test shows no statistically significant difference in specificity between the two methods (*p* = 0.549). **(D)** Overlap of positive detection results between mNGS and culture. Donut charts illustrating the concordance and discordance in positive pathogen detection between mNGS and culture methods across all 135 patients. The diagram highlights the number of cases detected exclusively by one method or by both.

Figure 4A shows the positive rates of mNGS and culture tests in both IG and NIG groups. The results of the mNGS method for the diagnosis of orthopedic infectious diseases were as follows: sensitivity of 94.06%, specificity of 85.29%, positive predictive value of 95.00%, negative predictive value of 82.86%, positive likelihood ratio of 6.39, and negative likelihood ratio of 0.07. The results of the clinical pathogenic microbial culture method were as follows: sensitivity of 47.52%, specificity of 76.47%, positive predictive value of 85.71%, negative predictive value of 32.91%, positive likelihood ratio of 2.02, and negative likelihood ratio of 0.69. Comparing the two methods, it can be seen in Figures 4B,C that the sensitivity of mNGS was improved by 46.54% (94.06% vs. 47.52%; *p* = 0.000), while the difference in specificity was not significant (85.29% vs. 76.47%; *p* = 0.549).

#### Concordance between mNGS and culture for pathogen detection

We further explored the concordance between the two results, as shown in Figure 4C, where 46 samples out of 135 cases were positive for both mNGS and culture (34.07%), and 25 samples (18.52%) were negative. A total of 54 samples were positive only for mNGS (40.00%), and 10 samples were positive only for culture (7.41%). In Figure 4D, of the samples that were positive on both cultures, the final reported results were an exact match for the microbial species in 17 of the 46 cases; however, 8 cases were an exact mismatch. The remaining 21 cases were “partly matched,” indicating overlap of at least 1 pathogen species.

#### “False positives” and “false negatives” of mNGS

In the IG group, as detailed in Table 2, 6 culturable pathogens were omitted by mNGS. Of these 6 “mNGS False Negatives” samples, 2 samples results were “Microbes Weak,” the mNGS outcome showed a low pathogen load, which we hypothesized was due to the patients’ own infection status, taking antibiotics, and poor sample selection, leading to low pathogen content in the sample sent for testing, and the pathogen nucleic acid was covered by the human nucleic acid information, which could not capture the relevant pathogen information, resulting in a false-negative result. And the other 4 were not recognized by mNGS at all; the result was contradictory to the final clinical judgment. After corresponding to the sample number in detail and recalling the sampling process, we found that it was caused by the poor quality of the sample, while the sample collection technique, the choice of sampling time, and the sample preservation were also common reasons affecting the mNGS result. After subsequent repetition of the mNGS test, the result showed a true positive. Meanwhile, Table 2 illustrates that potential explanations for the five “mNGS False Positives” in the NIG group include possible co-infection (2/5), over-interpretation (2/5), and unidentified factors (1/5).

**Table 2:** “False positives” and “false negatives” of mNGS.

Sample no.	Specimen source	Diagnosis	mNGS result	Possible explanation
Pathogens detected only by mNGS in the NIG group				
4	Fresh tissue	Rejection reaction	Malassezia, CMV	(1) Lack of clinical/radiological signs of fungal/viral infection; (2) Definitive alternative diagnosis, rejection; (3) No microbiological corroboration
10	Intraoperative pus swabbing method	Pathologic fractures	Enterococcus, CMV	(1) Lack of clinical signs supporting bacterial/viral co-infection; (2) No microbiological corroboration for either pathogen
17	Fresh tissue	Non-union	CMV	(1) Lack of clinical signs of viral infection; (2) No microbiological corroboration; (3) Definitive alternative diagnosis, non-union
20	Wound secretions	Rejection reaction	Candida smoothies	(1) Lack of clinical/radiological signs of fungal infection; (2) Definitive alternative diagnosis, rejection; (3) No microbiological corroboration
28	Intraoperative pus swabbing method	Inflammatory reaction	Phagocytic bacteria	(1) Lack of progressive clinical signs of infection; (2) No microbiological corroboration; (3) Clinical course consistent with aseptic inflammation.

Microbe	Count	Possible explanation
Culturable pathogens missed by mNGS in the IG group		
*Pseudomonas aeruginosa*	4	Positive not detected
Streptococcus	2	Microbes weak

### Comparison of mNGS and culture testing by pathogens and samples

#### Analysis and comparison of pathogen types and the sample-type level

**Figure 5:**
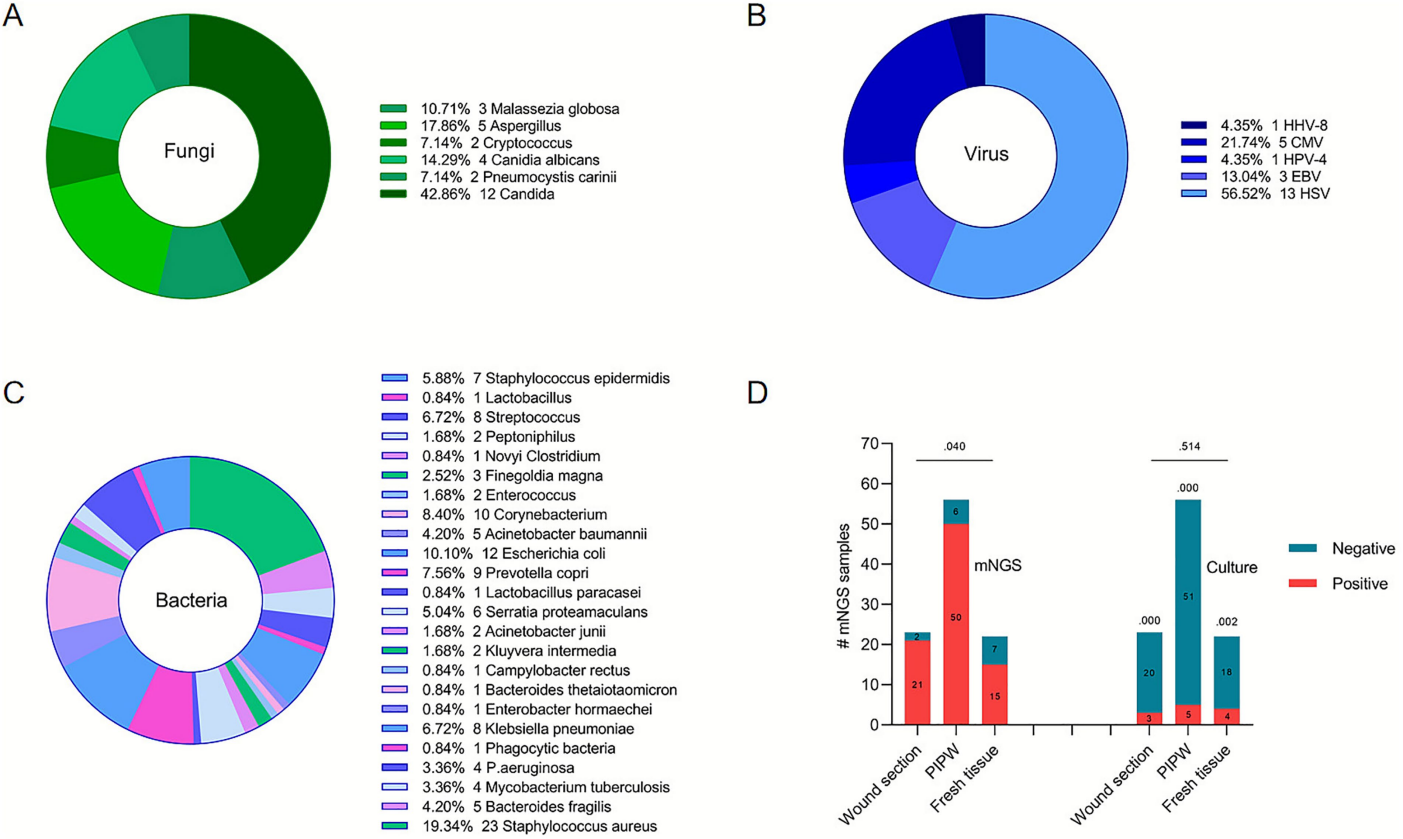
Bacterial, fungal, and viral species in mNGS and culture, and differences in the performance of the two assays in different sampling methods. **(A)** Distribution of fungal species identified by mNGS. Candida species were the most frequently detected fungi (42.86%). **(B)** Distribution of bacterial species identified by mNGS. *Staphylococcus aureus* was the most common bacterium (19.34%), followed by *Escherichia coli* and *Prevotella copri*. **(C)** Distribution of viral species identified by mNGS. Herpes simplex virus (HSV) accounted for the majority of viral detections (56.52%). **(D)** Comparison of sensitivity between mNGS and culture across three specimen types: wound secretions, intraoperative pus swabs, and fresh tissue. mNGS demonstrated significantly higher sensitivity than culture in all sample types (all *p* < 0.05), with overall sensitivity also differing significantly among specimen types (*p* = 0.040).

As shown in Figures 5A–C, *Staphylococcus aureus* (23/119) was the most common microorganism isolated from mNGS and culture test, followed by *Escherichia coli* (12/119), *Prevotella copri* (9/119), Streptococcus (8/119), and *Klebsiella pneumoniae* (8/119). Among the culture specimens, all 8 positive specimens were *Staphylococcus aureus* infections. *Klebsiella pneumoniae* (*n* = 8), *Pseudomonas aeruginosa* (*n* = 4), *Finegoldia magna* (*n* = 3), *Escherichia coli* (*n* = 2), Phagocytic bacteria (*n* = 1), and all fungi and viruses were detected only in mNGS-positive samples.

In the types of wound secretion, intraoperative purulent wiping and fresh tissue samples, the sensitivity of mNGS was significantly higher than that of culture (*p* = 0.000 for secretion, *p* = 0.000 for purulent fluid, *p* = 0.002 for tissue). At the same time, there was a significant difference in the overall sensitivity of mNGS among different types of specimens (*p* = 0.040) in Figure 5D.

### Correlative analysis between mNGS and laboratory results

#### Comparison of blood biochemical indices between infected and non-infected groups

All 135 patients were tested for infection-related indicators such as routine blood, CRP, and PCT on the day of pathogenic microbiological examination. In Table 3, the results of laboratory examination in different groups of patients showed that the WBC (*10^9^), Neutrophils (%), NEUT#, CRP, and PCT in the NIG were significantly lower than those in the IG group (*p* = 0.007, 0.014, 0.004, 0.011, and 0.046, respectively).

**Table 3:** Comparison of blood biochemical indices between infected and non-infected groups.

Laboratory parameters	IG	NIG	*P* value
WBC*10^9^	8.10 ± 1.33	6.75 ± 1.15	0.007
RBC*10^9^	4.06 ± 0.83	4.04 ± 0.68	0.910
Plt*10^9^	235.57 ± 50.44	227.26 ± 29.00	0.365
Hemoglobin, g/L	111.10 ± 20.57	108.20 ± 15.09	0.451
Neutrophils, %	64.57 ± 10.24	58.73 ± 9.70	0.014
NEUT#	6.19 ± 1.66	5.30 ± 1.10	0.004
Lymphocyte, %	27.10 ± 8.46	27.66 ± 8.15	0.336
LYM#	1.88 ± 0.0.83	1.72 ± 0.63	0.307
Monocyte, %	7.19 ± 1.87	6.94 ± 1.73	0.508
MONO#	0.63 ± 0.18	0.67 ± 0.20	0.262
CRP mg/L	47.24 ± 35.56	29.99 ± 27.62	0.011
PCT ng/mL	2.12 ± 1.00	1.73 ± 0.88	0.046

#### Comparison of clinical characteristics of mNGS-positive and mNGS-negative groups

Table 4 presents the comparison of clinical characteristics between the mNGS-positive and mNGS-negative groups, focusing on infection-related blood biochemistry indicators. In this study, peripheral blood indices, including leukocyte count, erythrocyte count, platelet count, CRP, and PCT levels, were assessed in both groups. Univariate analysis revealed that the leukocyte count, neutrophil count, and neutrophil percentage, as well as CRP and PCT concentrations, were significantly higher in the mNGS-positive group compared to the mNGS-negative group, with statistical significance (*p* = 0.037, 0.015, 0.033, 0.047, and 0.034, respectively). No statistically significant differences were observed between the groups for other measured indicators.

**Table 4:** The results of clinical characteristics of positive and negative groups by mNGS.

Laboratory parameters	Positive	Negative	*P* value
WBC*10^9^	8.23 ± 1.33	7.44 ± 1.10	0.037
RBC*10^9^	4.02 ± 0.84	4.39 ± 0.73	0.130
Plt*10^9^	238.86 ± 51.83	222.64 ± 36.93	0.264
Hemoglobin, g/L	112.06 ± 21.47	106.57 ± 15.05	0.360
Neutrophils, %	65.22 ± 10.59	58.92 ± 6.31	0.033
NEUT#	6.34 ± 1.69	5.17 ± 1.15	0.015
Lymphocyte, %	27.69 ± 8.84	25.19 ± 5.17	0.306
LYM#	1.89 ± 0.0.84	1.97 ± 0.59	0.744
Monocyte, %	7.27 ± 1.95	7.92 ± 2.35	0.271
MONO#	0.61 ± 0.17	0.71 ± 0.23	0.075
CRP mg/L	50.46 ± 36.37	30.09 ± 25.81	0.047
PCT ng/mL	2.26 ± 0.69	1.65 ± 0.83	0.034

#### Analysis of the relevant factors for mNGS positive group

To investigate the risk factors associated with positive mNGS test results in infected patients, a logistic multiple regression analysis was conducted, as shown in Table 5, to assess the correlation between blood biochemical indicators of infection. After adjusting for potential confounders, significant variables included leukocyte count, neutrophil percentage, neutrophil count, CRP, and PCT, all of which were positively associated with pathogen detection. The differences were statistically significant (*p* = 0.041, 0.036, 0.022, 0.040, and 0.046, respectively).

**Table 5:** The analysis of the relevant factors of mNGS positivity in patients.

Values	B	SE(B)	Wald χ^2^	*P* value	OR	95%CI
Age (years)	0.027	0.021	1.675	0.196	1.028	0.986, 1.072
Sex	−0.499	0.65	0.588	0.443	0.607	0.170, 2.173
WBC*10^9^	0.629	0.307	4.193	0.041	1.876	1.027, 3.426
Neutrophils, %	0.066	0.032	4.375	0.036	1.069	1.004, 1.137
NEUT#	0.662	0.288	5.276	0.022	1.939	1.102, 3.412
Lymphocyte, %	0.038	0.037	1.052	0.305	1.039	0.966, 1.117
LYM#	−0.114	0.346	0.109	0.742	0.892	0.453, 1.756
Monocyte, %	0.198	0.154	1.667	0.197	1.220	0.902, 1.648
MONO#	−2.763	1.587	3.031	0.082	0.063	0.003, 1.415
CRP mg/L	0.023	0.011	4.206	0.040	1.023	1.001, 1.046
PCT ng/mL	0.826	0.413	3.995	0.046	2.283	1.016, 5.130

### Potential implications of the clinical mNGS test

#### Potential inappropriate antibiotic usage for patients with virus isolates

In the IG group of 23 patients with viral infections, broad-spectrum antibiotics were initially administered based on clinical symptoms and imaging findings. However, in 9 cases, inappropriate antibiotic use was suspected, as the patients’ conditions did not improve or even worsened. mNGS was employed to identify the true pathogens, leading to the adjustment of antibiotic therapy, which subsequently resulted in clinical improvement (Table 6). Notably, after de-escalation of broad-spectrum antibiotics and initiation of targeted antiviral therapy guided by mNGS, clinical improvement was observed in 21 of the 23 patients (91.30%), demonstrating a high treatment success rate. To illustrate, one representative case involved a patient with suspected post-traumatic limb infection who did not respond to initial empiric carbapenems. mNGS of intraoperative pus identified Herpes Simplex Virus (HSV) as the predominant pathogen, with no significant bacterial sequences detected. Based on this finding, antibiotic therapy was discontinued, and intravenous acyclovir was initiated. The patient’s local signs of inflammation and systemic fever resolved within 72 h, confirming the viral etiology and underscoring the critical role of mNGS in averting unnecessary antibiotic exposure and guiding effective treatment.

**Table 6:** Clinical characteristics of patients with virus isolates (*N* = 23).

Virus	HAI		Broad-spectrum antibiotic		Inappropriate use of antibiotics		Treatment effectiveness after mNGS	
	Yes	No	Yes	No	Yes	No	Yes	No
CMV (*n* = 5)	3	2	5	0	2	3	5	0
HSV (*n* = 13)	11	2	8	5	4	9	11	2
HPV-4 (*n* = 1)	0	1	1	0	1	0	1	0
EBV (*n* = 3)	1	2	2	1	1	3	3	0
HHV-8 (*n* = 1)	0	1	1	0	1	0	1	0
Total (*N* = 23)	15	8	17	6	9	15	21	2

HAI, hospital-acquired infection; CMV, Cytomegalovirus; HSV, herpes simplex virus; HPV-4, human papillomavirus-4; EBV, Epstein–Barr virus; HHV-8, human herpes virus-8.

#### Case illustrations about mNGS-guided therapeutic adjustments for rare bacterial and polymicrobial infections

Beyond its impact on viral diagnosis, mNGS directly influenced antimicrobial therapy in cases involving rare bacteria and polymicrobial infections that were missed by conventional culture. We present two representative cases from our study to illustrate the translation of sequencing data into specific therapeutic actions, including antimicrobial selection, dosing, and treatment duration.

Case 1: Guidance for targeted therapy against a rare pathogen. A 65-year-old man presented with chronic osteomyelitis of the femur following internal fixation. Conventional bacterial and fungal cultures remained negative after 7 days. mNGS of intraoperative pus identified significant sequences of *Finegoldia magna*, a fastidious Gram-positive anaerobic coccus, with high genome coverage and strictly mapped reads far exceeding the background threshold. Based on this finding and the established susceptibility profile of *Finegoldia magna*, the antimicrobial regimen was deliberately de-escalated from empirical vancomycin to targeted therapy with intravenous clindamycin (600 mg every 8 h). The patient’s local inflammation and systemic fever resolved within 1 week. Given the chronic nature of osteomyelitis, targeted therapy was continued for a total duration of 6 weeks, resulting in successful clinical cure.

Case 2: Guidance for regimen escalation in polymicrobial infection. A 58-year-old woman with a diabetic foot infection, Wagner grade 3, had wound secretion samples sent for testing. Conventional culture only grew *Pseudomonas aeruginosa*. However, mNGS detected a polymicrobial community comprising *Escherichia coli*, *Prevotella copri*, and Candida glabrata. This comprehensive profile indicated a complex mixed aerobic-anaerobic-bacterial and fungal infection that was inadequately covered by the initial anti-pseudomonal cephalosporin. Consequently, the antibiotic therapy was escalated to meropenem (1 g every 8 h) to provide broader aerobic and anaerobic coverage, and micafungin was added for the Candida glabrata. The treatment duration was extended to 4 weeks with close monitoring, leading to significant wound improvement and avoidance of major amputation.

#### The influence of positive by mNGS on the hospital days, operation time, and rehospitalization rate of patients

In our study, there were 87 samples in the positive group with 75 men and 14 in the negative group with 9 men. There was no significant difference in mean age between the two groups (55.02 years vs. 53.93 years, *p* = 0.092). Patients in the mNGS-positive group exhibited a trend toward longer hospital stays (23.12 days vs. 21.79 days, *p* = 0.137) and a higher rehospitalization rate (66.85% vs. 50.00%, *p* = 0.032) compared to the mNGS-negative group, although these differences did not reach statistical significance (Table 7). At the same time, we compared the differences among different bone infection diseases, and the results showed that chronic osteomyelitis had the highest HOD, rehospitalization rate, and operation time (Table 8).

**Table 7:** The basic demographic and clinical characteristics of initial and outcome patient variables in mNGS.

Characteristics	Positive	Negative	*P* value
Sample amount	87	14	
Age (years)	55.02 ± 12.63	53.93 ± 17.70	0.092
Sex			
Male	75	9	0.057
Female	12	5	
Hospital day, median (IQR)	23.12 (20–29)	21.79 (18–25)	0.137
Rehospitalization rate, *n* (%)	58 (66.85%)	7 (50.00%)	0.032

**Table 8:** Comparison of the different bone infection diseases.

Clinical parameters	Osteomyelitis	Traumatic limb infections	Diabetic foot infections	Pressure ulcer	Spinal infection	Postoperative infection of the fracture	*P* value
Sample amount	55	28	7	6	2	3	
Median (IQR)							
Hospital day	22.37 (21–29)	21.81 (18–25)	21.97 (20–26)	20.45 (19–24)	21.45 (15–24)	23.12 (20–26)	0.417
Rehospitalization rate, *n* (%)	42 (76.40%)	11 (39.30%)	4 (57.08%)	2 (33.30%)	1 (50.00%)	2 (66.70%)	0.021
Operation time (min)	155 (125–190)	130 (95–175)	110 (105–115)	145 (138–153)	162 (161–163)	123 (122–124)	0.536

## Discussion

The traditional clinical model for diagnosing infectious diseases involves physicians making a differential diagnosis and conducting a series of tests to identify the pathogen. Traditional microbiological diagnostic techniques include smear microscopy, microbial culture, antigen–antibody detection, and PCR (29, 30). Bone infections can be classified into specific and non-specific infections. Specific bone infections are primarily caused by *Mycobacterium tuberculosis*, Brucella, *Treponema pallidum*, fungi, and others. Non-specific infections are commonly caused by *Staphylococcus aureus*, *Escherichia coli*, Streptococcus, and *Klebsiella pneumoniae*, with *Staphylococcus aureus* being the most prevalent. Due to microbial characteristics and drug resistance, treatment should be tailored to the specific pathogen. The indiscriminate use of antibiotics exacerbates dysbiosis and microbial resistance, hindering preoperative preparation and postoperative recovery. Therefore, antibiotic use in bone infection treatment is crucial, and rapid, accurate pathogen detection plays a vital role in treatment and prognosis. Early and precise use of antibiotics has become the key to changing the prognosis of bone infectious diseases, and the precise use of antibiotics cannot be separated from the precise judgment of pathogens that cause diseases.

Currently, bacterial culture remains the gold standard for diagnosing bone infections, though it has limitations, including low positivity rates and long turnaround times (31). Even when positive, delayed culture results may cause missed optimal treatment windows, affecting outcomes. Metagenomic next-generation sequencing (mNGS), also known as high-throughput sequencing (32), enables simultaneous sequencing of thousands to billions of DNA fragments (33), offering rapid, effective pathogen detection (34). Compared with the harsh culture conditions, long culture time, and low positive rate in the laboratory, mNGS has the advantages of being less susceptible to environmental interference, rapid, accurate, and with a high positive rate, which makes it suitable for the diagnosis of pathogens in bone infectious diseases. While widely used in infectious disease diagnosis and treatment, research on mNGS for bone infections is limited, and its diagnostic efficacy remains inconsistent. In this study, we analyzed the application and differences between traditional culture methods and mNGS in the diagnosis of clinical infectious diseases, and explored the application value of mNGS to provide an objective basis for clinical diagnosis and treatment. We collected wound secretions, intraoperative purulent swabs, and fresh tissue from 135 patients suspected of having bone infections and subjected these samples to both conventional microbiological assays and mNGS testing for a comparative analysis of their clinical features and diagnostic performance.

Bone infections, such as osteomyelitis, are common yet complex conditions in clinical practice, with chronic cases often exhibiting high incidence rates (35). The patient cohort in this study was representative, offering valuable insights for diagnosing and treating various types of infections. Our results demonstrated that there were no statistically significant differences in age, gender, length of hospital stay, or fatality between the study groups (*p* > 0.05), making the groups comparable. Compared to traditional culture methods, mNGS showed remarkably higher sensitivity in pathogen detection (36), particularly for complex infections that are difficult to diagnose via conventional culture. Although the specificity of mNGS (85.29%) was not significantly different from that of culture (76.47%), the marked improvement in pathogen detection rates underscores its diagnostic value in bone infections. However, the lower specificity of mNGS suggests a potential risk for false positives, necessitating cautious interpretation in conjunction with clinical symptoms and other diagnostic results. Furthermore, the relatively low concordance between mNGS and traditional culture highlights mNGS ability to detect pathogens that are missed by conventional methods and identify cases of mixed infections (37). Due to the discrepancies between these two approaches, further clinical validation is essential to minimize diagnostic errors and improve patient outcomes.

To further evaluate the diagnostic performance of metagenomic next-generation sequencing (mNGS), we conducted a comparative analysis with conventional microbial culture. The true-positive rate of mNGS was significantly higher than that of traditional culture methods (94.06% vs. 47.52%, *p* = 0.000). Moreover, mNGS demonstrated superior capacity in detecting fastidious or uncultivable pathogens, including novel bacteria, viruses, fungi, and atypical microorganisms. A study by Miao et al. (27) reported that the sensitivity of mNGS for diagnosing infectious diseases was 50.70%, notably higher than that of conventional cultures (50.70% vs. 35.20%). However, their study used traditional culture as the reference standard, which may have inflated the apparent false-positive rate of mNGS due to the inherently low positivity rate of culture. In contrast, our study adopted a comprehensive clinical diagnosis as the gold standard, enabling a more accurate assessment of the diagnostic discrepancies between mNGS and microbial culture. This approach effectively minimizes the impact of false positives and more faithfully reflects the diagnostic efficacy of mNGS. Our results confirm that mNGS possesses high sensitivity, supporting its utility in the early identification of pathogens and timely adjustment of antibiotic regimens. When used in conjunction with subsequent culture results, mNGS can contribute to the precision treatment of bone-related infections.

Besides, elevated serum biochemical markers were observed in patients with confirmed infections (*p* = 0.007, 0.014, 0.004, 0.011, and 0.046, respectively). Notably, the levels of white blood cells (WBC, 10^9^/L), neutrophil percentage, absolute neutrophil count (NEUT#), C-reactive protein (CRP), and procalcitonin (PCT), these biomarkers were also significantly higher in the mNGS-positive group compared to the mNGS-negative group (*p* = 0.037, 0.015, 0.033, 0.047, and 0.034, respectively), suggesting a strong concordance between mNGS results and clinical diagnostic outcomes. These findings indicate that early application of mNGS facilitates more timely and accurate identification of microbial infections, thereby contributing to improved patient management and guiding targeted antimicrobial therapy. This is particularly advantageous in the context of polymicrobial infections associated with specific diseases.

One of the key advantages of mNGS is that it does not require prior clinical knowledge to detect pathogens (38). This allows for rapid and accurate results, significantly reducing the time to diagnose infectious pathogens (39). The early and timely reporting of mNGS results can guide clinical decision-making, particularly in preventing the overuse of antibiotics for viral infections. Our result was evidenced by the high clinical success rate (91.30%) after therapy adjustment in patients with viral infections identified by mNGS. This aligns with the growing body of evidence demonstrating the impact of mNGS on antimicrobial stewardship. For instance, a large prospective multicenter study on febrile neutropenia in acute leukemia patients reported that 35.20% (81/218) of patients had their antimicrobial therapy adjusted based on mNGS results, and 97.50% (79/81) of those patients benefited clinically from the change (40). Our findings, consistent with these studies, reinforce that mNGS-driven pathogen detection facilitates more timely and targeted antimicrobial therapy, potentially improving patient outcomes and curbing antibiotic resistance. Additionally, mNGS has demonstrated utility in detecting rare and uncommon pathogens (41). It is capable of identifying anaerobes, fungi, and viruses that traditional culture methods fail to detect, enriching the pathogen spectrum for bone infections. This comprehensive detection capacity facilitates more accurate identification of complex microbial communities, particularly in cases of chronic or recurrent infections where multiple pathogens may be involved (42), providing crucial information for clinical treatment decisions.

Moreover, studies have shown that mNGS is not only effective for pathogen identification but also for microbiome characterization, host response analysis, drug resistance gene detection, and virulence factor identification (43). These capabilities have driven the rapid development and application of mNGS in difficult-to-diagnose cases involving immunocompromised patients or those with immunodeficiency. Another important advantage is that mNGS is less affected by prior antibiotic use (44), as pathogen DNA can remain detectable in plasma for an extended period. This is in contrast to traditional cultures, which are often compromised by previous antibiotic treatment (45). The higher sensitivity of mNGS observed in this study could be partly attributed to this reduced influence of antibiotic exposure. In our study, the observed diagnostic discrepancy between mNGS and culture, with an overall concordance of 52.59%, is a recognized phenomenon in the application of advanced molecular techniques to bone infectious diseases. This finding underscores the complementary nature of these methods and necessitates a careful exploration of the underlying reasons, which primarily revolve around the inherent limitations of conventional culture and the unprecedented sensitivity of mNGS. Several factors contribute to this culture failure: the prior administration of antimicrobial agents before sample collection can significantly suppress microbial growth while leaving detectable nucleic acid traces; the presence of fastidious, slow-growing, or intracellular pathogens, such as Mycobacteria, Brucella or anaerobes that have specific growth requirements not met by routine culture media; and the challenge of conventional techniques in accurately identifying polymicrobial infections, whereas mNGS can simultaneously detect all present genomes. In our results, the detection of viruses, fungi, and a broader spectrum of bacteria exclusively by mNGS supports this view. Besides, the heightened sensitivity of mNGS raises the legitimate question of whether it detects clinically irrelevant nucleic acids, originating from environmental contamination, sample colonization, or non-viable organisms. In our study, the specificity of mNGS remained high (85.29%) and was not statistically different from culture, which argues against widespread false positivity. However, we acknowledge this inherent challenge. The interpretation of mNGS results must always be contextualized within the clinical picture. To this end, we implemented stringent wet-lab procedures and predefined bioinformatic thresholds to minimize background noise (46, 47). Furthermore, for cases where mNGS was positive in the non-infection group, we applied a rigorous adjudication protocol based on clinical, microbiological, and treatment response criteria to differentiate potential false positives from true pathogens. This approach is supported by quality assessment studies, which indicate that a majority of false-positive signals in mNGS can be traced to laboratory contamination, underscoring the need for robust quality control. Therefore, while the detection of low-abundance microbial sequences is possible, the integration of these results with clinical symptoms, radiological findings, and other laboratory markers, such as elevated CRP and PCT in our mNGS-positive group, is paramount for accurate clinical decision-making (48). In conclusion, the discrepancy between mNGS and culture in diagnosing bone infections is not a mere technical artifact but rather a reflection of their complementary diagnostic philosophies. Culture, while highly specific, is constrained by its dependence on viable, cultivable organisms. In contrast, mNGS offers a culture-independent, panoramic view of the microbial landscape, which includes pathogens missed by culture but also requires expert interpretation to distinguish signal from noise. Our findings, consistent with other studies in the field, reinforce the value of mNGS as a powerful adjunct to traditional methods, particularly in culture-negative cases, complex infections, and for patients who have previously received antibiotics. It is imperative to interpret mNGS findings within the full clinical context, integrating them with the patient’s symptoms, radiological findings, laboratory inflammatory markers, such as the elevated CRP and PCT levels associated with mNGS-positive patients in our study and overall clinical course. Besides, the unbiased nature of mNGS allows for the detection of rare, fastidious, and unexpected pathogens, as well as the accurate characterization of polymicrobial communities. Our study demonstrated this through the identification of viruses, fungi, and anaerobes that were entirely missed by culture. The clinical value of this comprehensive detection is profound, as it directly enables more informed and precise antimicrobial therapy. As illustrated by our case examples, the detection of a rare anaerobe *Finegoldia magna* allowed for a targeted de-escalation from broad-spectrum coverage, while the uncovering of a complex polymicrobial infection necessitated an escalation and broadening of the regimen. This moves beyond empirical therapy and toward a tailored approach, influencing not only antimicrobial selection but also dosing strategies and guiding the duration of treatment based on the identified pathogens. The translation of mNGS data into therapeutic action, however, requires careful clinical correlation by a multidisciplinary team to differentiate true infection from contamination or colonization. This integrative approach is essential to harness the full potential of mNGS for improving patient outcomes and advancing antimicrobial stewardship.

We systematically compared mNGS and traditional culture methods in terms of sensitivity, specificity, pathogen types, and sample types. We also analyzed the differences between mNGS-positive and mNGS-negative groups. Patients in the mNGS-positive group tended to have worse prognoses, highlighting the need for closer clinical monitoring. In a word, patients who tested positive for mNGS had longer hospital stays and a higher rehospitalization rate, with no differences between male and female patients or age groups. However, the small sample size was a major limitation of our study, preventing some results from reaching statistical significance despite indicating certain trends. Future studies should include larger patient populations to improve the robustness of the findings. Our study has several limitations. First, this was an exploratory study, and a formal sample size calculation or power analysis was not conducted prior to patient enrollment. Therefore, it is possible that our study was underpowered to detect statistically significant differences for some outcomes, particularly for subgroup analyses. The findings should be interpreted as preliminary and require validation in larger, adequately powered prospective studies. Another limitation was the lack of randomization, as this was a retrospective study with data collection not controlled by the researchers. Other limitations include the single-center design, absence of a gold standard comparator for diagnostics, incomplete details on antibiotic usage, and potential classification bias. While mNGS has shown great promise in diagnosing clinical infections, it cannot yet fully replace traditional culture methods (49). Based on the findings of this study, we believe that mNGS should be considered a complementary tool rather than a complete substitute for culture in detecting pathogens in clinical bone infection samples. When cultures return negative or when hard-to-culture pathogens are suspected, mNGS can provide rapid and accurate diagnoses (50, 51), guiding adjustments to antibiotic regimens. Thought-provokingly, our study, consistent with others, demonstrates mNGS’s superior sensitivity in detecting a wide spectrum of pathogens, including viruses, fungi, and rare bacteria. However, this high sensitivity introduces the critical challenge of distinguishing true pathogens from background flora, contaminating sequences, or clinically insignificant colonizing microorganisms. The clinical relevance of a microorganism detected by mNGS is not inherent but must be interpreted systematically. Factors such as the microorganism’s known virulence, the sequence abundance, and the specimen type are crucial initial filters. Ultimately, the most definitive determinant is the integration of the mNGS result with the patient’s clinical context, including signs and symptoms of infection, radiological evidence, elevation of inflammatory markers as observed in our mNGS-positive group, and response to targeted therapy, a process often requiring multidisciplinary discussion. Consequently, when mNGS reports rare, unexpected, or low-confidence microorganisms, especially from potentially contaminated samples, it is strongly recommended to seek confirmation through orthogonal testing methods. These may include targeted PCR, offering higher sensitivity for specific pathogens, serological assays, or histopathological examination of tissue samples demonstrating inflammation and the presence of the microorganism. A limitation of this process is the lack of systematic confirmatory testing for every unusual mNGS finding, an aspect that should be incorporated into future prospective research designs to further solidify the etiological claims.

In sum, the study detected more potential pathogens through mNGS. However, due to the limitations of this method, these microorganisms may represent true infection agents or be the result of contamination, background flora, or skin colonization. Future research should focus on minimizing contamination during microbial sequencing, which remains one of the primary challenges facing mNGS methods today. Additionally, further studies should investigate drug resistance patterns and the clinical efficacy of treatments for these pathogens to better inform the use of antibiotics in clinical settings. For the diagnosis of bone infections, microbial compositions must be carefully interpreted in light of clinical symptoms and laboratory biochemical indices to ensure accurate diagnosis and treatment.

## Data Availability

The original contributions presented in the study are included in the article/supplementary material, further inquiries can be directed to the corresponding authors.

## References

[1] Baker RE Mahmud AS Miller IF Rajeev M Rasambainarivo F Rice BL . Infectious disease in an era of global change. Nat Rev Microbiol. (2021) 20:193–205. doi: 10.1038/s41579-021-00639-z, 34646006 PMC8513385

[2] Naghavi M Mestrovic T Gray A Gershberg Hayoon A Swetschinski LR Robles Aguilar G . Global burden associated with 85 pathogens in 2019: a systematic analysis for the global burden of disease study 2019. Lancet Infect Dis. (2024) 24:868–95. doi: 10.1016/S1473-3099(24)00158-0, 38640940 PMC11269650

[3] Seymour CW Gesten F Prescott HC Friedrich ME Iwashyna TJ Phillips GS . Time to treatment and mortality during mandated emergency Care for Sepsis. N Engl J Med. (2017) 376:2235–44. doi: 10.1056/NEJMoa1703058, 28528569 PMC5538258

[4] Jia C Wang X Yu S Wu H Shen J Huang Q . An antibiotic cement-coated locking plate as a temporary fixation for treatment of infected bone defects: a new method of stabilization. J Orthop Surg Res. (2020) 15:44. doi: 10.1186/s13018-020-1574-2, 32046768 PMC7014650

[5] Lu S Wang L Luo W Wang G Zhu Z Liu Y . Analysis of the epidemiological status, microbiology, treatment methods and financial burden of hematogenous osteomyelitis based on 259 patients in Northwest China. Front Endocrinol (Lausanne). (2023) 13:13. doi: 10.3389/fendo.2022.1097147, 36686458 PMC9846127

[6] Cooper AM Shope AJ Javid M Parsa A Chinoy MA Parvizi J. Musculoskeletal infection in pediatrics. Journal of bone and joint. Surgery. (2019) 101:e133. doi: 10.2106/JBJS.19.00572, 31567692

[7] Liu T Wang J Yuan Y Wu J Wang C Gu Y . Early warning of bloodstream infection in elderly patients with circulating microparticles. Ann Intensive Care. (2021) 11:110. doi: 10.1186/s13613-021-00901-w, 34255213 PMC8276897

[8] Qu X Wang S Qu Y Wang H Ye X Tang L . Antimicrobial susceptibility characteristics and risk factors associated with adult Sepsis in Wenzhou, China. Infect Drug Resist. (2022) 15:915–24. doi: 10.2147/IDR.S352570, 35299859 PMC8921831

[9] Nana A Nelson SB McLaren A Chen AF. What’s new in musculoskeletal infection: update on biofilms. J Bone Joint Surg. (2016) 98:1226–34. doi: 10.2106/JBJS.16.00300, 27440572

[10] Mayhew MB Buturovic L Luethy R Midic U Moore AR Roque JA . A generalizable 29-mRNA neural-network classifier for acute bacterial and viral infections. Nat Commun. (2020) 11:1177. doi: 10.1038/s41467-020-14975-w, 32132525 PMC7055276

[11] Radovanovic M Marthaler BR Nordstrom CW Petrovic M Dumic I Barsoum MK. *Cardiobacterium hominis* endocarditis incidentally diagnosed following an aortic valve replacement surgery. IDCases. (2022) 29:e01529. doi: 10.1016/j.idcr.2022.e01529, 35693329 PMC9184553

[12] Gu W Miller S Chiu CY. Clinical metagenomic next-generation sequencing for pathogen detection. Ann Rev Pathol Mech Dis. (2019) 14:319–38. doi: 10.1146/annurev-pathmechdis-012418-012751, 30355154 PMC6345613

[13] Schlaberg R Chiu CY Miller S Procop GW Weinstock G. Validation of metagenomic next-generation sequencing tests for universal pathogen detection. Arch Pathol Lab Med. (2017) 141:776–86. doi: 10.5858/arpa.2016-0539-RA, 28169558

[14] Li M Yang F Lu Y Huang W. Identification of *Enterococcus faecalis* in a patient with urinary-tract infection based on metagenomic next-generation sequencing: a case report. BMC Infect Dis. (2020) 20:467. doi: 10.1186/s12879-020-05179-0, 32615925 PMC7330266

[15] Wilson MR Naccache SN Samayoa E Biagtan M Bashir H Yu G . Actionable diagnosis of Neuroleptospirosis by next-generation sequencing. N Engl J Med. (2014) 370:2408–17. doi: 10.1056/NEJMoa1401268, 24896819 PMC4134948

[16] Besser J Carleton HA Gerner-Smidt P Lindsey RL Trees E. Next-generation sequencing technologies and their application to the study and control of bacterial infections. Clin Microbiol Infect. (2018) 24:335–41. doi: 10.1016/j.cmi.2017.10.013, 29074157 PMC5857210

[17] Chiu CY Miller SA. Clinical metagenomics. Nat Rev Genet. (2019) 20:341–55. doi: 10.1038/s41576-019-0113-7, 30918369 PMC6858796

[18] Rossen JWA Friedrich AW Moran-Gilad J. Practical issues in implementing whole-genome-sequencing in routine diagnostic microbiology. Clin Microbiol Infect. (2018) 24:355–60. doi: 10.1016/j.cmi.2017.11.001, 29117578

[19] Han D Li Z Li R Tan P Zhang R Li J. mNGS in clinical microbiology laboratories: on the road to maturity. Crit Rev Microbiol. (2019) 45:668–85. doi: 10.1080/1040841X.2019.1681933, 31691607

[20] Deng X Achari A Federman S Yu G Somasekar S Bártolo I . Author correction: metagenomic sequencing with spiked primer enrichment for viral diagnostics and genomic surveillance. Nat Microbiol. (2020) 5:525–5. doi: 10.1038/s41564-020-0671-7, 31965087 PMC7608365

[21] Liu Y Wu W Xiao Y Zou H Hao S Jiang Y. Application of metagenomic next-generation sequencing and targeted metagenomic next-generation sequencing in diagnosing pulmonary infections in immunocompetent and immunocompromised patients. Frontiers in cellular and infection. Microbiology. (2024) 14:14. doi: 10.3389/fcimb.2024.1439472 PMC11333343 39165919

[22] Greninger AL Naccache SN Federman S Yu G Mbala P Bres V . Rapid metagenomic identification of viral pathogens in clinical samples by real-time nanopore sequencing analysis. Genome Med. (2015) 7:99. doi: 10.1186/s13073-015-0220-9, 26416663 PMC4587849

[23] Wang C Hu J Gu Y Wang X Chen Y Yuan W. Application of next-generation metagenomic sequencing in the diagnosis and treatment of acute spinal infections. Heliyon. (2023) 9:e13951. doi: 10.1016/j.heliyon.2023.e13951, 36879954 PMC9984843

[24] Jeon YJ Zhou Y Li Y Guo Q Chen J Quan S . The feasibility study of non-invasive fetal trisomy 18 and 21 detection with semiconductor sequencing platform. PLoS One. (2014) 9:e110240. doi: 10.1371/journal.pone.0110240, 25329639 PMC4203771

[25] Li H Durbin R. Fast and accurate short read alignment with burrows-wheeler transform. Bioinformatics. (2009) 25:1754–60. doi: 10.1093/bioinformatics/btp324, 19451168 PMC2705234

[26] Duan H Li X Mei A Li P Liu Y Li X . The diagnostic value of metagenomic next-generation sequencing in infectious diseases. BMC Infect Dis. (2021) 21:62. doi: 10.1186/s12879-020-05746-5, 33435894 PMC7805029

[27] Miao Q Ma Y Wang Q Pan J Zhang Y Jin W . Microbiological diagnostic performance of metagenomic next-generation sequencing when applied to clinical practice. Clin Infect Dis. (2018) 67:S231–40. doi: 10.1093/cid/ciy693, 30423048

[28] Jiang X Yan J Huang H Ai L Yu X Zhong P . Development of novel parameters for pathogen identification in clinical metagenomic next-generation sequencing. Front Genet. (2023) 14:1266990. doi: 10.3389/fgene.2023.1266990, 38046047 PMC10693447

[29] Tao Y Song L Fu H Zhang W Song Y Liu H . Application of microbiological rapid on-site evaluation in respiratory intensive care units: a retrospective study. Ann Transl Med. (2022) 10:7–7. doi: 10.21037/atm-21-5465, 35242852 PMC8825533

[30] Wang M Liu W Xiong Z Li Z Li J Xu X . Case report: “area of focus” atypical Trichinellosis and fascioliasis coinfection. Front Med. (2022) 9:9. doi: 10.3389/fmed.2022.881356, 35646994 PMC9132012

[31] Ding J Ma B Wei X Li Y. Detection of Nocardia by 16S ribosomal RNA gene PCR and metagenomic next-generation sequencing (mNGS). Frontiers in cellular and infection. Microbiology. (2022) 11:11. doi: 10.3389/fcimb.2021.768613, 35071035 PMC8779735

[32] Xu H Hu X Wang W Chen H Yu F Zhang X . Clinical application and evaluation of metagenomic next-generation sequencing in pulmonary infection with pleural effusion. Infect Drug Resist. (2022) 15:2813–24. doi: 10.2147/IDR.S365757, 35677528 PMC9167844

[33] Gan Z Liu J Wang Y Yang L Lou Z Xia H . Performance of metagenomic next-generation sequencing for the diagnosis of Cryptococcal meningitis in HIV-negative patients. Frontiers in cellular and infection. Microbiology. (2022) 12:12. doi: 10.3389/fcimb.2022.831959, 35531340 PMC9069553

[34] Guo X Zhang X Qin Y Liu H Wang X. Endocarditis due to *Aggregatibacter Segnis*: a rare case report. BMC Infect Dis. (2023) 23:309. doi: 10.1186/s12879-023-08231-x, 37158846 PMC10169330

[35] Qiao Y Liu X Li B Han Y Zheng Y Yeung KWK . Treatment of MRSA-infected osteomyelitis using bacterial capturing, magnetically targeted composites with microwave-assisted bacterial killing. Nature. Communications. (2020) 11:4446. doi: 10.1038/s41467-020-18268-0, 32895387 PMC7477539

[36] Gu W Deng X Lee M Sucu YD Arevalo S Stryke D . Rapid pathogen detection by metagenomic next-generation sequencing of infected body fluids. Nat Med. (2020) 27:115–24. doi: 10.1038/s41591-020-1105-z, 33169017 PMC9020267

[37] Liu C Chen Q Fu P Shi Y-Y. Anncaliia algerae Microsporidiosis diagnosed by metagenomic next-generation sequencing. China Emerg Infect Dis. (2022) 28:1466–70. doi: 10.3201/eid2807.212315, 35731183 PMC9239868

[38] He Y Fang K Shi X Yang D Zhao L Yu W . Enhanced DNA and RNA pathogen detection via metagenomic sequencing in patients with pneumonia. J Transl Med. (2022) 20:195. doi: 10.1186/s12967-022-03397-5, 35509078 PMC9066823

[39] Deng B Hua J Zhou Y Zhan D Zhu L Zhan Y . Legionella pneumonia complicated with rhabdomyolysis and acute kidney injury diagnosed by metagenomic next-generation sequencing: a case report. World J Emerg Med. (2023) 14:322. doi: 10.5847/wjem.j.1920-8642.2023.063, 37425079 PMC10323505

[40] Feng S Rao G Wei X Fu R Hou M Song Y . Clinical metagenomic sequencing of plasma microbial cell-free DNA for febrile neutropenia in patients with acute leukaemia. Clin Microbiol Infect. (2024) 30:107–13. doi: 10.1016/j.cmi.2023.05.034, 37271194

[41] Chen T Zhang L Huang W Zong H Li Q Zheng Y . Detection of pathogens and antimicrobial resistance genes in ventilator-associated pneumonia by metagenomic next-generation sequencing approach. Infect Drug Resist. (2023) 16:923–36. doi: 10.2147/IDR.S397755, 36814827 PMC9939671

[42] Huang W Han D Cai Q Yi X Tang J Fang Y . First identification of human infection with Erysipelothrix Piscisicarius by metagenomic next-generation sequencing. Emerg Microbes Infect. (2022) 11:2781–4. doi: 10.1080/22221751.2022.2140614, 36287140 PMC9662008

[43] Xie X Xi X Zhao D Zhao Y Yi T Chen D . Advancing pathogen and tumor copy number variation detection through simultaneous metagenomic next-generation sequencing: a comprehensive review. Heliyon. (2024) 10:e38826. doi: 10.1016/j.heliyon.2024.e38826, 39568836 PMC11577201

[44] Zhang S Wu G Shi Y Liu T Xu L Dai Y . Understanding etiology of community-acquired central nervous system infections using metagenomic next-generation sequencing. Frontiers in cellular and infection. Microbiology. (2022) 12:12. doi: 10.3389/fcimb.2022.979086, 36225235 PMC9549810

[45] Molina FJ Botero LE Isaza JP Cano LE López L Tamayo L . Diagnostic concordance between BioFire® FilmArray® pneumonia panel and culture in patients with COVID-19 pneumonia admitted to intensive care units: the experience of the third wave in eight hospitals in Colombia. Crit Care. (2022) 26:130. doi: 10.1186/s13054-022-04006-z, 35534867 PMC9084542

[46] Guo Y Yang Y Xu M Shi G Zhou J Zhang J . Trends and developments in the detection of pathogens in central nervous system infections: a bibliometric study. Frontiers in cellular and infection. Microbiology. (2022) 12:12. doi: 10.3389/fcimb.2022.856845, 35573778 PMC9100591

[47] Nan X Zhang Y Su N Yang L Pan G. Application value of metagenomic next-generation sequencing for bloodstream infections in pediatric patients under intensive care. Infect Drug Resist. (2022) 15:1911–20. doi: 10.2147/IDR.S357162, 35465251 PMC9031986

[48] Zhan Y Cao J Ji L Zhang M Shen Q Xu P . Impaired mitochondria of Tregs decreases OXPHOS-derived ATP in primary immune thrombocytopenia with positive plasma pathogens detected by metagenomic sequencing. Exp Hematol Oncol. (2022) 11:48. doi: 10.1186/s40164-022-00304-y, 36050760 PMC9434515

[49] Gu L Liu W Ru M Lin J Yu G Ye J . The application of metagenomic next-generation sequencing in diagnosing *Chlamydia psittaci* pneumonia: a report of five cases. BMC Pulm Med. (2020) 20:65. doi: 10.1186/s12890-020-1098-x, 32178660 PMC7077129

[50] Yang S Xue B Hu X Zhou W Zhang M Zhao M. Spinal infection caused by *Coxiella burnetii*. BMC Infect Dis. (2023) 23:6. doi: 10.1186/s12879-022-07938-7, 36609227 PMC9817394

[51] Hai L Li P Xiao Z Zhou J Xiao B Zhou L. Rhizopus microsporus and Mucor racemosus coinfection following COVID-19 detected by metagenomics next-generation sequencing: a case of disseminated mucormycosis. Heliyon. (2024) 10:e25840. doi: 10.1016/j.heliyon.2024.e25840, 38370187 PMC10869847

